# Visceral adiposity index is associated with silent brain infarct in a healthy population

**DOI:** 10.1038/s41598-020-74454-6

**Published:** 2020-10-14

**Authors:** Ki-Woong Nam, Hyung-Min Kwon, Han-Yeong Jeong, Jin-Ho Park, Hyuktae Kwon, Su-Min Jeong, Hyun-Jin Kim

**Affiliations:** 1 Departments of Neurology, Seoul National University College of Medicine and Seoul National University Hospital, Seoul, South Korea; 2 Department of Neurology, Seoul National University College of Medicine and Seoul Metropolitan Government-Seoul National University Boramae Medical Center, 20 Boramae-ro 5-gil, Dongjak-gu, Seoul, 07061 South Korea; 3 Departments of Family Medicine, Seoul National University College of Medicine and Seoul National University Hospital, 101 Daehak-ro, Jongno-gu, Seoul, 03080 South Korea; 4 Departments of Family Medicine, Seoul National University College of Medicine and Seoul Metropolitan Government-Seoul National University Boramae Medical Center, Seoul, South Korea; 5 National Cancer Control Institute, National Cancer Center, Goyang, South Korea

**Keywords:** Stroke, Neurology, Cerebrovascular disorders, White matter disease, Obesity

## Abstract

Visceral adiposity index (VAI) has been associated with various cardio-metabolic diseases; however, there is limited information about its association with cerebrovascular diseases. In this study, we evaluated the relationship between VAI and silent brain infarct (SBI). We evaluated a consecutive series of healthy volunteers over the age of 40 between January 2006 and December 2013. SBI was defined as an asymptomatic, well-defined lesion with a diameter ≥ 3 mm with the same signal characteristics as the cerebrospinal fluid. VAI was calculated using sex-specific equations as described in previous studies. A total of 2596 subjects were evaluated, and SBI was found in 218 (8%) participants. In multivariable analysis, VAI (adjusted odds ratio [aOR] = 1.30; 95% confidence interval [CI] 1.03–1.66; *P* = 0.030) remained a significant predictor of SBI after adjustment for confounders. The close relationship between VAI and SBI was prominent only in females (aOR = 1.44; 95% CI 1.00–2.07; *P* = 0.048). In the evaluation between VAI and the burden of SBI, VAI showed a positive dose–response relationship with the number of SBI lesions (*P* for trend = 0.037). High VAI was associated with a higher prevalence and burden of SBI in a neurologically healthy population.

## Introduction

Global obesity rates are currently increasing^1^, leading to serious health complications worldwide that include cardio/cerebrovascular diseases, atherosclerosis, and subclinical inflammation^1,2^. However, such obesity-related complications do not occur in all obese patients and vary depending on individual characteristics^3,4^. In recent years, it has been recognized that the distribution of fat plays a substantially more important role in obesity-related complications than dose the amount of total fat^5–7^. Visceral adipose tissue (VAT) has been shown to be associated with the risk of insulin resistance and cardio/cerebrovascular disease^6,8,9^, whereas subcutaneous adipose tissue (SAT) appears to be less influential and even rather protective^6,10^. Therefore, instead of using the traditional body mass index (BMI), various indicators reflecting fat distribution differences across individuals have been developed and used.

For accurate measurements of the distribution and amount of fat, imaging modalities such as magnetic resonance imaging (MRI) and computed tomography (CT) have been used^11,12^. However, as shown in recent studies on “metabolically healthy obesity”, in addition to problems with quantity of adipose tissue, functional aspects such as the presence of accompanying metabolic diseases must be considered^1,4^. On this theoretical basis, Amato et al. developed a sex-specific index for predicting VAT mass and function using waist circumference (WC), BMI, triglyceride (TG), and high-density lipoprotein (HDL) cholesterol ^8^. This visceral adiposity index (VAI) was closely associated with metabolic disease, cardiovascular disease, and even atherosclerosis^10–14^. However, studies on cerebrovascular disease is still lacking.

Silent brain infarct (SBI) is a subclinical pathological condition that is commonly found in elderly individuals^15,16^. SBI itself is asymptomatic but increases the risk of subsequent strokes^16^. Therefore, a clear understanding of the pathological mechanisms and risk factors of SBI may be helpful in the primary prevention of cerebrovascular disease in the subclinical stage. In this study, we evaluated the relationship between VAI and SBI in a neurologically healthy population to gain insight into underlying pathological mechanisms.

## Results

A total of 2596 neurologically healthy participants (mean age 56 ± 7 years, male sex: 54%) were included. SBI was found in 218 (8%) participants, and the mean VAI was 1.66 ± 1.33. Other detailed baseline characteristics are presented in Supplementary Table 1.

**Table 1:** Univariate analysis between with SBI and without SBI groups.

	No SBI (n = 2408)	SBI (n = 188)	*P* value
Age, n (%)			< 0.001
< 55 year	1115 (46)	36 (19)	
55–64 year	1014 (42)	86 (46)	
≥ 65 year	279 (12)	66 (35)	
Sex, male, n (%)	1301 (54)	99 (53)	0.717
Body mass index, kg/m^2^ [IQR]	23.95 [22.16–25.83]	23.97 [22.04–26.03]	0.642
Hypertension, n (%)	504 (21)	67 (36)	< 0.001
Diabetes, n (%)	312 (13)	39 (21)	0.002
Ischemic heart disease, n (%)	67 (3)	5 (3)	0.921
Current smoking, n (%)	438 (18)	31 (16)	0.560
Use of antiplatelet agents, n (%)	189 (8)	26 (14)	0.004
Use of antihypertensives, n (%)	431 (18)	56 (30)	< 0.001
Use of glucose-lowering agents, n (%)	122 (5)	12 (6)	0.432
Systolic BP, mmHg [IQR]	125 [115–136]	131 [120–142]	< 0.001
Diastolic BP, mmHg [IQR]	75 [69–83]	78 [72–87]	< 0.001
Hemoglobin A1c, % [IQR]	5.7 [5.5–6.0]	5.9 [5.5–6.2]	< 0.001
Fasting glucose, mmol/L [IQR]	5.01 [4.62–5.50]	5.06 [4.68–5.94]	0.058
Total cholesterol, mmol/L [SD]	5.23 ± 0.92	5.14 ± 0.94	0.139
LDL cholesterol, mmol/L [IQR]	3.34 [2.74–3.88]	3.21 [2.53–3.98]	0.352
HDL cholesterol, mmol/L [IQR]	1.38 [1.17–1.64]	1.33 [1.14–1.57]	0.151
Triglyceride, mmol/L [IQR]	1.11 [0.82–1.62]	1.18 [0.86–1.68]	0.147
White blood cell, × 10^3^/μL [IQR]	5.24 [4.34–6.30]	5.35 [4.34–6.30]	0.367
hs-CRP, mg/dL [IQR]	0.04 [0.01–0.15]	0.06 [0.01–0.17]	0.262
Homocysteine, μmol/L [IQR]	9.5 [8.1–11.5]	9.6 [8.1–11.8]	0.919
VAT, cm^2^ [IQR]*	106.87 [72.99–148.74]	121.25 [82.86–161.59]	0.008
SAT, cm^2^ [IQR]*	150.59 [114.56–196.01]	149.23 [118.29–201.53]	0.332
TAT, cm^2^ [IQR]*	265.37 [206.67–332.56]	284.11 [223.61–340.70]	0.035
Visceral adiposity index, [IQR]	1.26 [0.84–2.01]	1.44 [0.90–2.31]	0.037

*SBI* silent brain infarct, *BP* blood pressure, *LDL* low-density lipoprotein, *HDL* high-density lipoprotein, *hs-CRP* high-sensitivity C-reactive protein, *VAT* visceral adipose tissue, *SAT* subcutaneous adipose tissue, *TAT* total adipose tissue.

*These variables were measured in 2136 participants.

**Figure 1:**
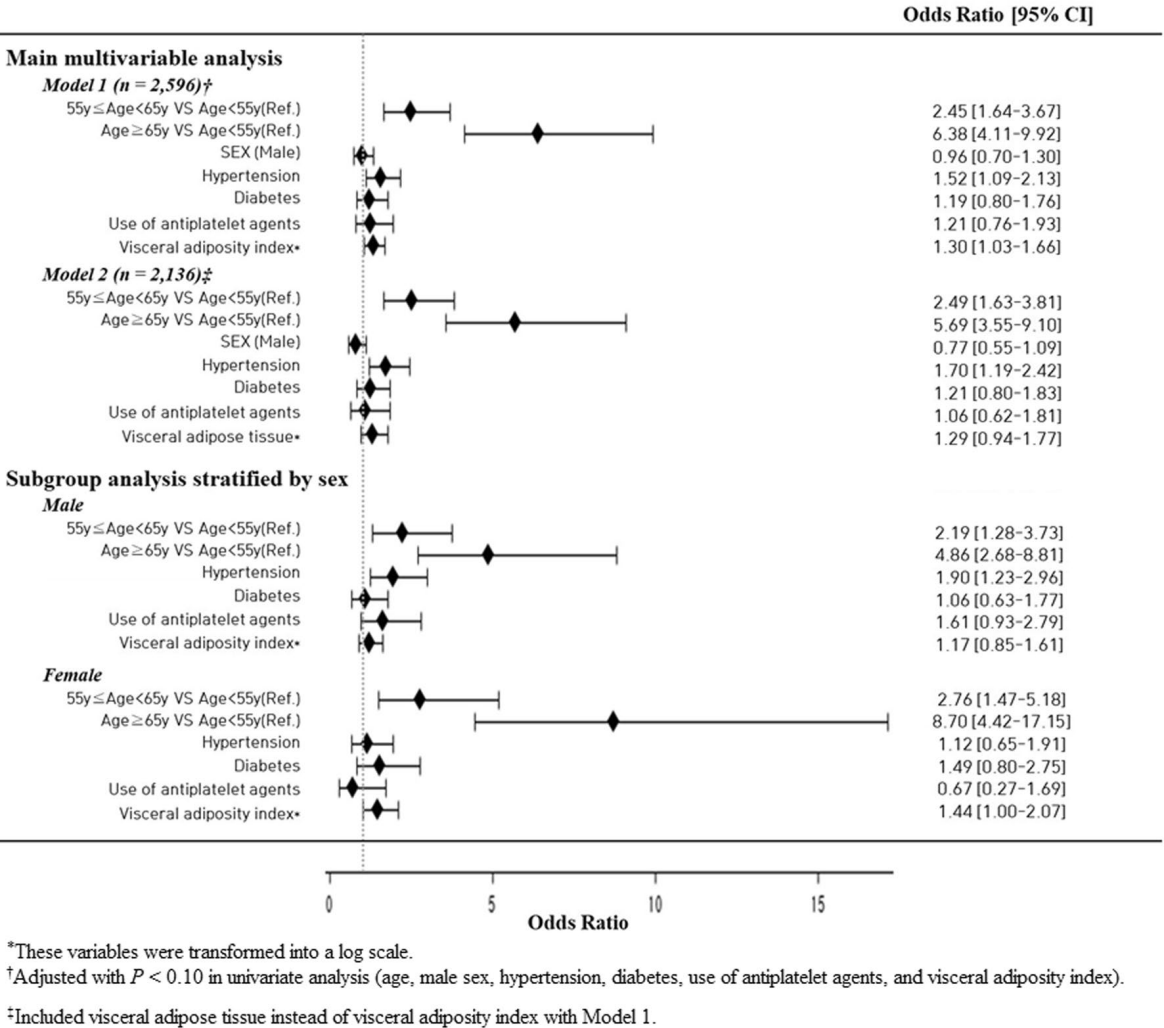
Multivaraible logistic regression analysis of possible predictors for silent brain infarct and subgroup analysis stratified by sex. In multivariable analysis, VAI (adjusted odds ratio [aOR] = 1.30; 95% confidence interval [CI] 1.03–1.66; *P* = 0.030) was associated with SBI after adjustment for confounders. On the other hand, VAT did not show any statistically significant association with SBI (aOR = 1.29; 95% CI 0.94–1.77; *P* = 0.121). This close relationship was prominent only in females (aOR = 1.44; 95% CI 1.00–2.07; *P* = 0.048), while no statistical significance was found in males.

**Figure 2:**
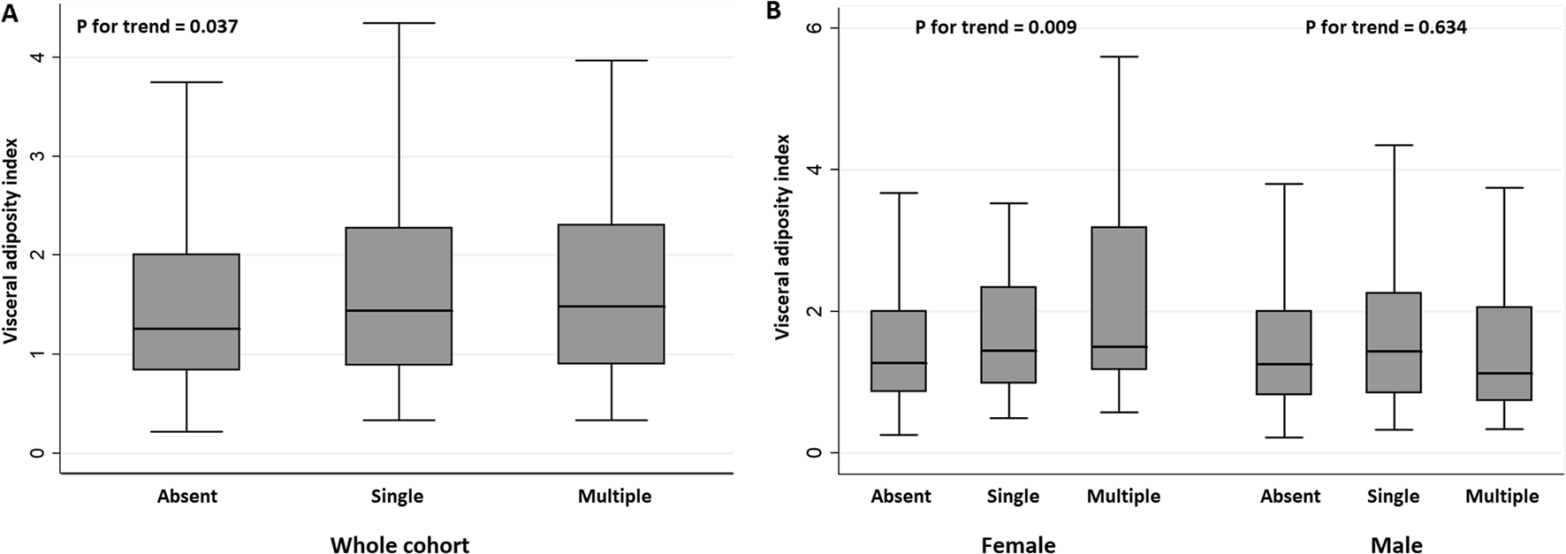
Distribution of median visceral adiposity index (VAI) according to the burden of silent brain infarct (SBI). Patients with multiple SBI lesions had a higher VAI as compared to those with absent or a single lesion in a dose–response manner (*P* for trend = 0.037). This tendency was more pronounced in female participants (*P* for trend = 0.009) and not in male participants (*P* for trend = 0.643).

Univariate analysis showed that SBI was significantly associated with age, hypertension, diabetes, use of antiplatelet agents and antihypertensives, systolic and diastolic BP, hemoglobin A1c level, VAT, TAT, and VAI (Table 1). In a multivariable logistic regression analysis to find possible predictors for SBI, VAI remained significant after adjusting for confounders [adjusted odds ratio (aOR) = 1.30; 95% confidence interval (CI) 1.03–1.66; *P* = 0.030]. Age and hypertension were also positively associated with SBI, independent of VAI (Fig. 1). VAI even showed a dose–response relationship according to the number of SBI lesions (*P* for trend = 0.037) (Fig. 2). On the other hand, VAT did not show any statistically significant association with SBI (aOR = 1.29; 95% CI 0.94–1.77; *P* = 0.121) (Fig. 1).

The close relationship between VAI and SBI was prominent only in females, both in their prevalence (aOR = 1.44; 95% CI 1.00–2.07, *P* = 0.048) and burden (*P* for trend = 0.009) (Figs. 1 and 2). Meanwhile, no statistical significance was found in male participants. These sex differences can be interpreted based on the results shown in Table 2. For most risk factors, similar results were found between male and female participants, but VAI was more strongly associated with hypertension or diabetes in female participants. Notably, males had a negative correlation between VAI and age, whereas females had a positive correlation (Table 2).

**Table 2:** Univariate linear regression analysis between visceral adiposity index^*^ and risk factors.

	Total	Male	Female
	β (95% CI)	β (95% CI)	β (95% CI)
Age			
< 55 year	Ref	Ref	Ref
55–64 year	− 0.049 (− 0.102 to 0.005)	− 0.159 (− 0.235 to − 0.084)	0.080 (0.006 to 0.154)
≥ 65 year	− 0.032 (− 0.109 to 0.046)	− 0.213 (− 0.322 to − 0.104)	0.188 (0.079 to 0.297)
Male sex	− 0.027 (− 0.077 to 0.022)	–	–
Hypertension	0.128 (0.068 to 0.187)	0.101 (0.019 to 0.183)	0.173 (0.085 to 0.261)
Diabetes	0.206 (0.133 to 0.278)	0.170 (0.076 to 0.264)	0.293 (0.177 to 0.410)
Ischemic heart disease	− 0.028 (− 0.180 to 0.123)	− 0.112 (− 0.317 to 0.094)	0.098 (− 0.127 to 0.323)
Current smoking	0.265 (0.201 to 0.329)	0.353 (0.278 to 0.428)	0.049 (− 0.137 to 0.234)
HbA1c*	0.973 (0.757 to 1.189)	0.765 (0.492 to 1.037)	1.485 (1.120 to 1.850)
Fasting glucose*	0.743 (0.617 to 0.869)	0.601 (0.436 to 0.766)	1.104 (0.900 to 1.308)
Total-c	0.072 (0.046 to 0.099)	0.096 (0.057 to 0.135)	0.045 (0.009 to 0.082)
LDL-c	0.052 (0.021 to 0.083)	0.042 (− 0.001 to 0.086)	0.063 (0.020 to 0.106)
White blood cell	0.096 (0.081 to 0.110)	0.111 (0.091 to 0.130)	0.089 (0.066 to 0.112)
hs-CRP*	0.069 (0.053 to 0.085)	0.072 (0.049 to 0.095)	0.068 (0.044 to 0.092)
Homocysteine*	0.147 (0.030 to 0.264)	0.278 (0.091 to 0.465)	0.235 (0.045 to 0.425)
VAT*	0.438 (0.395 to 0.482)	0.516 (0.453 to 0.579)	0.458 (0.394 to 0.523)
SAT*	0.332 (0.276 to 0.387)	0.438 (0.360 to 0.516)	0.255 (0.163 to 0.346)
TAT*	0.523 (0.465 to 0.580)	0.569 (0.494 to 0.644)	0.446 (0.355 to 0.536)
SBI	0.112 (0.016 to 0.208)	0.034 (− 0.104 to 0.173)	0.198 (0.066 to 0.329)
Number of SBI	0.057 (0.000 to 0.114)	0.002 (− 0.085 to 0.090)	0.104 (0.030 to 0.178)

*HbA1c* hemoglobin A1c, *Total-c* total cholesterol, *LDL-c* low-density lipoprotein cholesterol, *hs-CRP* high-sensitivity C-reactive protein, *VAT* visceral adipose tissue, *SAT* subcutaneous adipose tissue, *TAT* total adipose tissue, *SBI* silent brain infarct.

*These variables were transformed into a log scale.

The reason why the direction of association between age, the most powerful risk factor for SBI, and VAI was apposite in male and female can be explained by the apposite change of anthropometric indices level as aging in both sex (Table 3). Male participants showed a decrease in anthropometric indices (e.g., BMI and SAT) as they grew older, while an increase was seen in females (e.g., BMI, WC, VAT, and TAT) (Table 3).

**Table 3:** Difference of anthropometric indices according to age in male and female participants.

	55 > Age	65 > Age ≥ 55	Age ≥ 65	*P* for trend
**Male**	639	573	188	
BMI	24.70 [23.13–26.42]	24.34 [22.58–25.94]	24.19 [22.06–25.87]	< 0.001
WC	89.0 [84.0–93.0]	88.5 [83.0–93.5]	89.3 [84.0–94.0]	0.695
VAT	127.60 [91.01–167.11]	127.92 [91.51–165.42]	139.80 [92.28–181.54]	0.233
SAT	136.12 [106.70–173.49]	127.82 [96.79–160.70]	127.66 [100.62–161.39]	0.003
TAT	266.78 [210.32–333.56]	258.88 [199.97–322.87]	271.36 [202.36–342.20]	0.361
VAI	1.44 [0.87–2.21]	1.21 [0.80–1.87]	1.08 [0.76–1.85]	< 0.001
**Female**	512	527	157	
BMI	23.03 [21.06–24.76]	23.45 [21.79–25.37]	23.64 [21.74–25.65]	0.001
WC	80.3 [75.0–85.8]	84.0 [79.0–89.0]	85.5 [80.0–91.5]	< 0.001
VAT	74.09 [53.01–98.62]	91.58 [67.56–122.41]	110.53 [78.66–155.74]	< 0.001
SAT	176.49 [138.85–218.78]	184.68 [143.85–229.26]	182.81 [139.66–227.81]	0.128
TAT	255.61 [197.56–316.11]	277.46 [226.26–348.43]	293.87 [235.23–370.34]	< 0.001
VAI	1.19 [0.83–1.85]	1.32 [0.90–2.08]	1.48 [0.98–2.19]	< 0.001

*BMI* body mass index, *WC* waist circumference, *VAT* visceral adipose tissue, *SAT* subcutaneous tissue, *TAT* total adipose tissue, *VAI* visceral adiposity index.

## Discussion

In this study, we found that high VAI level was associated with both SBI prevalence and burden in a neurologically healthy population, while VAT area was not. Our findings suggest that VAI which encompasses metabolic susceptibility caused by visceral adiposity is more relevant predictor for SBI than simple VAT quantity. These meaningful findings were found only in female participants, consistent with previous studies^13,17,18^.

Although the exact pathological mechanisms underlying the relationship between VAI and SBI is not clear. Several plausible mechanisms can be considered. First, this may be due to synergistic effects of well-known risk factors included in the VAI calculation formula ^8^. High TG, low HDL cholesterol, and high WC are all well-known cerebrovascular risk factors^19–21^. Thus, the VAI developed from these combinations may be more strongly associated with SBI than for each individual risk factor. Second, the effects of metabolic comorbidities cannot be ignored. The aforementioned lipid profiles exhibited by high VAI are also called “atherogenic dyslipidemia”, a common finding in visceral obesity, insulin resistance, and metabolic syndrome^12,22^. Indeed, in both previous studies and our findings (Table 2), VAI has shown to be closely associated with metabolic diseases such as hypertension and diabetes, each of which is a major risk factor for SBI^8–10,14,23^. Third, endothelial dysfunction due to subclinical inflammation may be the main cause. Adipose tissue is not just a storage organ that actively releases various hormones or substances^6^. In particular, VAT tends to secrete more pro-inflammatory adipokines (e.g., IL-6, IL-8, TNF-a, and PAI-1) than SAT^12,23–25^. Therefore, subclinical inflammation might be severe in patients with high VAI^5^, which mainly reflects the function of VAT. This could be partly confirmed by the close relationship between VAI and WBC, hs-CRP, homocysteine in Table 2. Endothelial dysfunction in small arteries/arterioles may cause their occlusion or block perivascular glymphatic drainage, resulting in SBI development^15,26^. Lastly, high VAI can indicate an underlying higher atherosclerosis burden. Previous studies have shown that VAI is associated with intra- and extracranial atherosclerosis^3,5,13^, probably due to the aforementioned atherogenic dyslipidemia or subclinical inflammation. Advanced atherosclerotic lesions can develop SBI with chronic diffuse hypoperfusion and extravasation of toxic metabolites into neural tissues^15,26^.

Perhaps, the most interesting finding in this study is that even though VAI was created by adjusting for sex differences^8^, it is meaningful only in females. Previous studies investigating atherosclerosis or cardiovascular diseases have shown similar results, but the cause of these results is not clear^13,17,18^. They speculate that the hormonal difference between males and females or differences in composition of VAT and SAT underlie these results^17^. Based on our findings, we propose a slightly different reason. Firstly, the relationship between age and VAI may affect this sexual difference. Age is the strongest risk factor for cerebrovascular disease, including SBI. As shown in Tables 2 and 3, there is no significant increase in VAT with age in male patients, but rather a negative relationship between VAI and age with decreasing SAT. On the other hand, as females get older, anthropometric indicators (e.g., WC, VAT, and TAT) increase markedly and VAI increases. As a result, in females, VAI also serves as an indicator of age and may have more pronounced results. In addition, females had a lower prevalence of metabolic disease, but association between metabolic disease and VAI was stronger than in males (Table 2 and Supplementary Table 1). In other words, visceral obesity is more likely to be involved in the development of potent risk factors such as hypertension and diabetes, and there are fewer other confounders that can influence VAI’s impact on SBI. This may make VAI more influential in females.

In addition, our study presents two more interesting insights. First, VAI had a stronger predictive power on the prevalence of SBI than VAT. This may indicate that functional activity is more important than the amount of visceral fat^5,10,14^, and is consistent with recent recognition that metabolic status is more important than the severity of obesity^4^. Second, the cut-off value of VAI considering sensitivity and specificity for SBI prediction in our data was 1.22. The value is lower than the cut-off value of previous studies about the relationship with metabolic syndrome or cardiovascular disease^10,14^, but similar to those dealing with subclinical atherosclerosis^12,13^. These results indicate that subclinical pathology is also being carried out in patients with a VAI value of less than 2.00 which was thought to be traditional “normal range”, and that screening brain MRI and MRA may be required.

There are several caveats to interpret our findings. First, the current study is a retrospective observational study. We included a relatively homogeneous and large population; however, the possibility of selection bias still remains. Second, due to the limitations of cross-sectional analysis, we could only prove the association, not causality. Further prospective studies are needed to confirm our results. Third, because we only included a healthy population, the prevalence of SBI and cardiovascular risk factors were relatively small. Therefore, the results of this study cannot be easily generalized and applied to clinical fields. However, despite the small number of outcome events, VAI and SBI showed a strong relationship.

We demonstrated that high VAI level is associated with SBI prevalence and burden in a neurologically healthy population, especially in females. VAI can be calculated from the results of simple laboratory examinations commonly used in the clinical fields, and its predictive power for SBI is superior to VAT in our data. Thus, our findings indicate that VAI could be used as a simple and convenient predictive marker for the prevalence and burden of SBI.

## Methods

### Patients and population

From a consecutive registry of health check-ups at Seoul National University Hospital Health Promotion Center between January 2006 and December 2013, we retrospectively included subjects over the age of 40 years (n = 2904). Among them, 49 participants who had a history of stroke or clinically meaningful neurological deficit were excluded. Participants using lipid lowering agents (n = 219) and having missing data regarding covariates (n = 40) were also excluded. Finally, a total of 2596 neurologically healthy participants were included in the final cross-sectional analyses.

This retrospective cross-sectional study was approved by the Institutional Review Board (Number: H-1502-026-647) at Seoul National University Hospital. Requirement of informed consent from participants was waived by the Institutional Review Board of Seoul National University Hospital due to the retrospective study design using anonymized information. All experiments were performed in accordance with the Declaration of Helsinki and relevant guidelines and regulations.

### Clinical assessment

As part of routine health check-ups, we evaluated demographic factors, clinical factors, and cardiovascular risk factors in all participants. Age, sex, BMI, hypertension, diabetes, ischemic heart disease, current smoking, use of medications (e.g., antiplatelet agents, antihypertensives, and glucose lowering agents), systolic and diastolic blood pressure (BP) were assessed^4,6^. Laboratory examinations were conducted after 12 h overnight fasting, including glucose profiles, lipid profiles, white blood cell (WBC) counts, high-sensitivity C-reactive protein (hs-CRP) and homocysteine levels^4,6^.

The VAI was calculated using the sex-specific equations as described in previous studies: VAI = [WC/(39.68 + 1.88 × BMI)] × (TG/1.03) × (1.31/HDL cholesterol) for males and VAI = [WC/(36.58 + 1.89 × BMI)] × (TG/0.81) x (1.52/HDL cholesterol) for females^8^.

### Radiological assessment

All participants underwent brain MRI using 1.5-T MR scanners (SIGNA [GE Healthcare, Milwaukee, WI, USA] or Magnetom SONATA [Siemens, Munich, Germany]). The detailed descriptions of MRI acquisitions were as follows: basic slice thickness, 5 mm; T1-weighted images, repetition time (TR)/echo time (TE) = 500/11 ms; T2-weighted images, TR/TE = 5000/127 ms; T2-gradient echo images, TR/TE = 57/20 ms; and T2 fluid-attenuated inversion recovery images, TR/TE = 8800/127 ms. SBI was defined as asymptomatic, well-defined lesions, > 3 mm in size with the same signal characteristics as cerebrospinal fluid on T1 and T2 images^4,15^. We also rated the burden of SBI as absent, single, or multiple, based on their number^4^.

In this dataset, most participants (n = 2136) conducted abdominal fat CT scans using a 16-detector row CT scanner (Somatom Sensation 16, Siemens Medical Solutions, Forchheim, Germany)^6^. To obtain the adipose tissue area, we used a technique that we have previously validated^6^. We first obtained a single slice at the umbilicus level at 5 mm thickness with a scan time of 0.5 s. Then, we calculated the cross-sectional surface area (in cm^2^) of the different abdominal compartment using commercially available CT software (Rapidia 2.8; INFINITT, Seoul, Korea)^6^. The adipose tissue area was determined electronically using the setting of attenuation levels from -250 to -50 Hounsfield units. VAT was identified as the intra-abdominal fat that was surrounded by the parietal peritoneum or transversalis fascia, and excluding the vertebral column and paraspinal muscles. SAT was calculated by subtracting the VAT from the total adipose tissue (TAT) area^6^.

### Statistical analysis

We performed all statistical analyses using SPSS version21.0 (IBM SPSS, Chicago, IL, USA). Univariate analyses for assessing possible predictors for SBI were performed using Student’s *t*-test or Mann–Whitney *u*-test for continuous variables and Chi-squared test or Fisher’s exact test for categorical variables. Variables with severely skewed data were transformed into a log scale. Based on the results of univariate analysis, variables with *P* < 0.10 were introduced into the multivariable logistic regression analysis. We wondered how the VAI was related to the burden as well as to the prevalence of SBI, and compared the median VAI values according to the SBI burden using the Jonckheere–Terpstra test (*P* for trend.). In addition, we conducted additional sensitivity analyses to compare the predictive power between VAI and VAT on fat CT.

Males and females differ in composition of VAT and SAT^6^. Thus, VAI was formulated differently for males and females in consideration of sex differences^8^. Nevertheless, previous studies have shown that VAI is more influential in females^13,17,18^. We, therefore, conducted a stratified multivariable analysis by sex to confirm these results. All variables with *P* < 0.05 were considered significant in this study.

## Supplementary information

Supplementary Table.
