# Supplementary material for: Visceral adiposity index is associated with silent brain infarct in a healthy population
Source: Sci Rep. 2020 Oct 14;10:17271. doi: 10.1038/s41598-020-74454-6 (PMC7566629; doi:10.1038/s41598-020-74454-6)
Supplement: Supplementary file 1 — Supplementary Table. [file 41598_2020_74454_MOESM1_ESM.docx]

**Supplemental information**

**Visceral Adiposity Index is Associated with Silent Brain Infarct in a Healthy Population**

Ki-Woong Nam, MD, MSc^1^; Hyung-Min Kwon, MD, PhD^2,*^; Han-Yeong Jeong, MD^1^; Jin-Ho Park, MD, MPH, PhD^3,*^; Hyuktae Kwon, MD, PhD^3^; Su-Min Jeong, MD^4^; Hyun-Jin Kim, PhD^5^

**Supplementary Table 1. Baseline characteristics of the cohort (n = 2,596)**

|  | Total  (n = 2,596) | Male  (n = 1,400) | Female  (n = 1,196) |
| --- | --- | --- | --- |
| Age, n (%) | 56 ± 7 | 55 ± 7 | 56 ± 7 |
| < 55y | 1,151 (44) | 639 (46) | 512 (43) |
| 55-64y | 1,100 (42) | 573 (410 | 527 (44) |
| ≥ 65y | 345 (13) | 188 (13) | 157 (13) |
| Sex, male, n (%) | 1400 (54) | … | … |
| Body mass index, kg/m^2^ | 24.12 ± 3.01 | 24. 59 ± 2.85 | 23.57 ± 3.10 |
| Hypertension, n (%) | 571 (22) | 346 (25) | 225 (19) |
| Diabetes, n (%) | 351 (14) | 237 (17) | 114 (10) |
| Ischemic heart disease, n (%) | 514 (20) | 266 (19) | 248 (21) |
| Current smoking, n (%) | 469 (18) | 426 (30) | 43 (4) |
| Use of antiplatelet agents, n (%) | 215 (8) | 142 (10) | 73 (6) |
| Use of antihypertensives, n (%) | 487 (19) | 298 (21) | 189 (16) |
| Use of glucose lowering agents, n (%) | 134 (5) | 97 (7) | 37 (3) |
| Systolic blood pressure, mmHg | 126 ± 15 | 128 ± 15 | 124 ± 16 |
| Diastolic blood pressure, mmHg | 76 ± 11 | 78 ± 11 | 74 ± 10 |
| Hemoglobin A1c, % | 5.9 ± 0.8 | 5.9 ± 0.9 | 5.8 ± 0.6 |
| Fasting glucose, mmol/L | 5.30 ± 1.27 | 5.46 ± 1.46 | 5.11 ± 0.97 |
| Total cholesterol, mmol/L | 5.22 ± 0.93 | 5.13 ± 0.90 | 5.34 ± 0.94 |
| LDL cholesterol, mmol/L | 3.34 ± 0.89 | 3.32 ± 0.88 | 3.37 ± 0.90 |
| HDL cholesterol, mmol/L | 1.42 ± 0.36 | 1.33 ± 0.33 | 1.53 ± 0.36 |
| Triglyceride, mmol/L | 1.34 ± 0.83 | 1.49 ± 0.94 | 1.17 ± 0.64 |
| White blood cell, x 10^3^/μL | 5.52 ± 1.67 | 5.86 ± 1.75 | 5.12 ± 1.48 |
| hs-CRP, mg/dL | 0.18 ± 0.69 | 0.20 ± 0.72 | 0.15 ± 0.65 |
| Homocysteine, μmol/L | 10.1 ± 3.3 | 11.3 ± 3.5 | 8.5 ± 2.4 |
| Visceral adipose tissue, cm^2*^ | 118.39 ± 74.40 | 137.28 ± 80.74 | 95.29 ± 58.02 |
| Subcutaneous adipose tissue, cm^2*^ | 163.62 ± 86.40 | 140.51 ± 70.12 | 191.87 ± 95.59 |
| Total adipose tissue, cm^2*^ | 282.01 ± 140.81 | 277.79 ± 139.98 | 287.16 ± 141.72 |
| Visceral adiposity index | 1.66 ± 1.33 | 1.66 ± 1.35 | 1.65 ± 1.30 |
| Silent brain infarct, n (%) |  |  |  |
| Single | 141 (5) | 66 (6) | 75 (5) |
| Multiple | 47 (2) | 23 (2) | 24 (2) |

LDL = low-density lipoprotein, HDL = high-density lipoprotein, hs-CRP = high-sensitivity C-reactive protein

^*^These variables were measured in 2,136 participants.
